# Huangkui capsules regulate tryptophan metabolism to improve diabetic nephropathy through the Keap1/Nrf2/HO-1 pathway

**DOI:** 10.3389/fphar.2025.1535352

**Published:** 2025-04-30

**Authors:** Jiayu Su, Ying Zhang, Xuan Wang, Xiaochao Hu, Ke Zhou, Huimin Zhu, Ehu Liu, Shijia Liu

**Affiliations:** ^1^ The Affiliated Hospital of Nanjing University of Chinese Medicine, Jiangsu Province Hospital of Chinese Medicine, Nanjing, Jiangsu, China; ^2^ College of Pharmacy, China Pharmaceutical University, Nanjing, Jiangsu, China; ^3^ College of Pharmacy, Nanjing University of Chinese Medicine, Nanjing, Jiangsu, China

**Keywords:** diabetic nephropathy, huangkui capsules, oxidative stress, tryptophan metabolism, Keap1/ Nrf2/HO-1 pathway

## Abstract

**Background:**

Diabetic nephropathy (DN) is a serious complication of diabetes and one of the leading causes of end-stage renal disease. Huangkui capsule (HKC), a traditional Chinese patent medicine, is widely used in clinical practice for the treatment of chronic glomerulonephritis. However, the therapeutic effects and underlying mechanisms of HKC in DN remain poorly understood.

**Methods:**

DN was induced in db/db mice, which were randomly divided into the DN, HKC-L, HKC-H and IRB groups, and db/m mice served as the Control group. Biochemical indices of blood and urine samples from the mice were measured, and HE staining, Masson staining and PAS staining were used to verify the anti-DN effect of HKC. The levels of ROS and the expression of Nrf_2_ pathway-related proteins and mRNAs were detected. Metabonomic analysis was used to investigate the role of tryptophan metabolism in the regulation of DN by HKC. HK-2 cells were used to establish a model of high-glucose (HG) injury *in vitro*, and HKC treatment was given for supplementary verification. Sarpogrelate hydrochloride (SH) combined with HKC, a 5-HT_2A_R inhibitor, was used to verify the effect of the 5-HT pathway in an *in vitro* model.

**Results:**

Treatment with HKC significantly inhibited the increase in blood glucose and Urinary albumin/creatinine ratio (UACR), improved kidney injury signs in mice, reduced the level of ROS and improved oxidative stress injury through the Keap1/ Nrf_2_/HO-1 pathway. Metabonomic analysis revealed that tryptophan metabolism is involved in the process by which HKC improves DN, and HKC can regulate the 5-HT pathway to improve the renal injury by oxidative stress regulation. HKC treatment also significantly improved the renal and oxidative stress injuries in HG HK-2 cell model through the Nrf_2_ pathway *in vitro*. SH administration revealed that inhibiting 5-HT_2A_R could significantly inhibit the synthesis of 5-HT and improve the renal injury induced by HG.

**Conclusion:**

Our study demonstrate that HKC can inhibit kidney injury and oxidative stress injury in db/db mice and HK-2 cells by regulating tryptophan metabolism and the Keap1/Nrf_2_/HO-1 pathway, which provides new insight for the clinical use of HKC for treatment of DN.

## 1 Introduction

Persistent high blood glucose can result in a range of complications, such as cardiovascular issues, nerve damage, and kidney impairment. Diabetic nephropathy (DN), a frequent complication in type 2 diabetes mellitus patients, is characterized by renal vessel damage due to hyperglycemia and renal system malfunction (Gregg et al., 2014; ALBVR et al., 2019). The clinical symptoms of DN include renal function impairment, proteinuria, glomerular hypertrophy, fibrosis, and reduced filtration capacity, contributing significantly to diabetes-related mortality (DeFronzo et al., 2015). Nowadays, the standard treatments for DN include angiotensin II receptor antagonists, sodium-glucose cotransporter 2 inhibitors, and PLP-1R agonists. However, the multifaceted nature of DN and the absence of definitive intervention targets have hindered the treatment breakthroughs, leading to an increase in the number of patients with end-stage DN. Current treatments can delay disease progression but not prevent progression to end-stage renal disease (ESRD), which often requires prolonged therapy and poses the risk of severe adverse effects and recurrence (Palmer et al., 2015).

Traditional Chinese medicines (TCMs) offer distinct advantages in treating chronic, polygenic complex conditions; it acts through multiple components, targets, and pathways to address various disease stages, restores body homeostasis, and achieves lasting therapeutic effects. Huangkui capsule (HKC), an ethanol extract prepared from *Abelmoschus manihot* (L.) medic., is rich in flavonoids and is commonly used for chronic glomerulonephritis, IgA nephropathy, diabetic nephropathy, and nephrotic syndrome, demonstrating its wide efficacy (Zhang et al., 2014). Its potential mechanisms in renal disease treatment include anti-inflammatory, antioxidant, immunomodulatory, and renal tubular epithelial cell-protective effects as well as improvements in podocyte apoptosis, glomerular sclerosis, mesangial proliferation, and renal fibrosis inhibition (Li et al., 2021). Studies by Han et al. revealed that HKC could mitigate renal tubular epithelial mesenchymal transition by inhibiting NLRP3 inflammasome activation and renal TLR4/NF-κB signaling (Han et al., 2019). Gu et al. reported that when combined with metformin, HKC could inhibit the Klotho/TGF-β1/p38MAPK signaling pathway, reduce renal fibrosis both *in vivo* and *in vitro* (Gu et al., 2021). Mao et al. demonstrated that HKC could alleviate oxidative stress and renal fibrosis in DN rat models via the p38MAPK/Akt pathway, providing renal protection (Mao et al., 2015). However, further research into the specific mechanisms involved in this treatment is necessary.

Renal damage in diabetic nephropathy is primarily due to the increased oxidative stress, which impacts metabolic processes, pathways, and hemodynamics. In diabetic patients, hyperglycemia triggers increased production of reactive oxygen species (ROS), further exacerbating oxidative stress. Nuclear factor erythroid 2-related factor 2 (Nrf_2_) is a stress-responsive transcription factor, which is the downstream of ROS (Ma, 2013). During oxidative stress, Nrf_2_ is translocated to the nucleus to activate the transcription of genes reliant on antioxidant response elements (AREs) for ROS elimination and the maintenance of the cellular redox balance (Kobayashi and Yamamoto, 2005). Heme oxygenase-1 (HO-1), a pivotal antioxidant enzyme, is regulated by Nrf_2_ (Loboda et al., 2016). The upregulation of Nrf_2_ has been reported to confer protection in diabetic nephropathy animal models (Xie et al., 2018; Qiao et al., 2019). Nrf_2_ and its endogenous inhibitor Keap1 serve as intracellular defenses against oxidative stress. Under normal conditions, Nrf_2_ is bound to cytoplasmic Keap1 and targeted for proteasomal degradation. In the presence of oxidative stress, Nrf_2_ dissociates from Keap1 and migrates to the nucleus. The activation of the downstream gene HO-1 modulates the progression of various chronic diseases by regulating oxidative stress and lipid peroxidation (Bellezza et al., 2018).

5-HT, or 5-hydroxytryptamine, also known as serotonin, is a naturally occurring vasoactive agent involved in glucose and lipid metabolism, vascular inflammation, and atherosclerosis (Katz et al., 1994). In the periphery, tryptophan is hydroxylated by tryptophan hydroxylase 1 (Tph1) and then decarboxylated by aromatic amino acid decarboxylase (AADC) to yield 5-HT (El-Merahbi et al., 2015). Studies indicate that 5-HT plays a crucial regulatory role in the renal system. Excessive activation of peripheral 5-HT may be associated with the onset of diabetic nephropathy (Doggrell, 2003a). The function of 5-HT is contingent upon its receptors, which are categorized into seven families (1–7 5-HT receptors). The 5-HT_2_ receptor (5-HT_2_R) encompasses the subtypes 5-HT_2A_R, 5-HT_2B_R, and 5-HT_2C_R, which are widely distributed in organs such as the stomach, intestine, heart, kidneys, and adipose tissue (Yang et al., 2017a). Research has shown that SH, a 5-HT_2A_R antagonist, can effectively enhance renal function and mitigate pathological changes in diabetic nephropathy (Hoyer et al., 2002). 5-HT_2A_R is expressed in proximal renal tubular cells (Lee et al., 2017), and Tph1 and AADC are also present in proximal renal tubular epithelial cells (Xu et al., 2007; Hafdi et al., 1996).

This investigation aims to elucidate the effects of HKC in ameliorating renal injury in DN and to demonstrate that modulating the Keap1/Nrf_2_/HO-1 pathway and the 5-HT pathway may play an important role in DN treatment.

## 2 Materials and methods

### 2.1 HKC preparation and reagents

HKC was produced by Jiangsu Suzhong Pharmaceutical Group (Jiangsu China). One HKC was 0.43 g. Irbesartan was purchased from Zhejiang Huahai Pharmaceutical Group (Zhejiang China). Sarpogrelate hydrochloride was purchased from Ambeed (Chicago, United States, A166685). Detection kits for superoxide dismutase (SOD) (article number: ADS-W-KY011), glutathione (GSH) (article number: ADS-W-G001) and malondialdehyde (MDA) (article number: ADS-W-YH002) were obtained from Jiangsu Addison Biotechnology Co., Ltd., (Jiangsu China).

### 2.2 Animal and experimental protocols

Six-week-old male db/db mice (weighing approximately 45 ± 2 g) and db/m mice (weighing approximately 20 ± 2 g) were purchased from the Experimental Animal Center at Nanjing University of Chinese Medicine. The mice were kept in an SPF animal facility, and all the animals received humane care and had free access to water and food. The temperature was 23°C ± 2°C, the humidity was 60% ± 10%, and the light and dark cycle was alternated for 12 h. All the mice were allowed to adapt to the environment 1 week before the formal experiment. All the experimental procedures were implemented according to the Animal Ethics Committee of Nanjing University of Chinese Medicine (No. 202401A085). After 1 week of adaptive feeding, the mice in the control group (Control group, n = 6) were db/m mice, and the other four groups were db/db mice. They were permitted to eat normally and drink normally. Db/db mice were randomly divided into four intervention groups: the model group (DN group, n = 6), HKC low-dose treatment group 0.75 g/kg/d (HKC-L group, n = 6), HKC high-dose group 1.50 g/kg/d and irbesartan group 45 mg/kg/d. The drug and carrier were given by oral gavage every day for 8 weeks, and the mice were killed at the end of the eighth week.

### 2.3 Determination of biochemical indicators

During the experiment, the weights of the mice were measured once a week using an electronic balance. Urine and blood samples were collected after 8 weeks of HKC treatment. The blood and urine samples were incubated at room temperature for 1.5 h and then centrifuged at 4°C and 3,000 rpm for 15 min to obtain the supernatant. The samples were subsequently centrifuged at 4°C, 2,000 rpm, and 10 min to obtain the supernatant, which was stored at −80°C for later use. Blood and urine samples were measured with an automatic biochemical analyzer in the laboratory at Jiangsu Provincial Hospital of Traditional Chinese Medicine. The detection indices included blood glucose (Glu), total cholesterol (TC), triglyceride (TG), low-density lipoprotein cholesterol (LDL-C), high-density lipoprotein cholesterol (HDL-C), urea nitrogen (BUN), serum creatinine (SCR) and urinary albumin (UALB), Urine Creatinine (UCR), Urine Albumin/Urinary Creatinine (UACR) and Urine Specific Gravity (USG).

### 2.4 Pathological staining analysis

The renal tissue was fixed, embedded and sliced. Hematoxylin-eosin (HE) staining, Masson staining and periodic acid-Schiff (PAS) staining were performed. The standard indices of HE staining are as follows: (1) the number of cells in renal tubules and mesangial hyperplasia; (2) the degeneration/ necrosis of renal tubular epithelial cells; (3) interstitial inflammatory cell infiltration; and (4) medullary interstitial fibrosis. According to the severity of various lesions, the semi-quantitative score mild, 0.5; mild, 1; moderate, 2; severe, 3; extremely severe, 4; and nonpathological, 0. According to the degree of increase in the number of collagen fiber components in the renal interstitium, a small number of “1” and “2” fibers are increased, which obviously increases, the local interstitium is obviously widened by “3”, and the number of collagen fiber components in most renal interstitia increased by “4”. PAS staining score standard: According to the degree of basement membrane thickening and matrix increase in the glomerulus, there is no “0”, a small amount of “1”, “2”, which is obviously increased, and the local mesangium is obviously widened by “3”.

### 2.5 Intrarenal ROS measurements

The frozen kidney tissue sections were heated and incubated with DHE diluted in PBS in the dark at 37°C for 30 min. Then DAPI dye solution was added dropwise and dyed in the dark for 10 min at room temperature. Finally, the film was sealed by anti-fluorescence quenching method. Observe the slices and collect images under Nikon inverted fluorescence microscope. The intensity of fluorescence signal was analyzed by ImageJ software version 2.0.0.

### 2.6 Cell culture and treatment

The human kidney epithelial cell line HK-2 (Human Kidney-2) was cultured in MEM supplemented with 10% fetal bovine serum (FBS) supplemented with two antibodies at 37°C and 5% CO_2_. HK-2 cells were mixed with 60 mM glucose to establish a HG model, and HKC suspensions (100, 300,500 μg/mL) were subsequently cultured for 24 h, after which the cells were collected for further analysis.

### 2.7 Real-time fluorescence quantitative PCR

Total RNA was isolated using a total RNA extraction kit (19211ES60, YEASEN, Shanghai, China), and cDNA was prepared by reverse transcription with the HiScript II QRT Supermix for qPCR (^+^GDNa Wiper) (R233, Vazyme, Nanjing, China). According to the manufacturer’s instructions, ChamQ Blue Universal SYBR qPCR Master Mix (Q312, Vazyme, Nanjing, China) was used for RT-qPCR. The specificity and detection efficiency of the primer sequences were examined by standard curve analysis. The base sequence is shown in Supplementary Table S1.

### 2.8 Western blotting assay

The kidney tissue or cells were lysed on ice for 30 min using cell lysis buffer, the supernatant was centrifuged at 4°C and 12,000 rpm for 10 min, 1× loading buffer was added, and the mixture was incubated in a metal bath at 100°C for 10 min. The obtained protein samples were stored at −80°C until further analysis. Electrophoresis was performed with 10% SDS-PAGE, and the proteins were transferred from the gel to an NC membrane. After the membrane was blocked with 5% skim milk for 1 h, various primary antibodies were added, and the mixture was incubated at 4°Covernight. The primary antibodies used here included α-SMA (T55027, 1:1,000, Abmart, Shanghai, China), vimentin (T55134F, 1:1,000, Abmart, Shanghai, China), Nrf_2_ (80593-1-RR, 1:8,000, Proteintech, Wuhan, China) and Keap1 (60027-1-Ig, 1:3,000, Proteintech, Wuhan, China), HO-1 (10701-1-AP, 1:3,000, Proteintech, Wuhan, China), AADC (A3828, 1:1,000, ABclonal, Wuhan, China), 5-HT_2A_R (PA1564S, 1:1,000, Abmart, Shanghai, China), AHR (67785-1-Ig, 1:5,000, Proteintech, Wuhan, China), and CYP1A1 (A2159, 1:1,000, ABclonal, Wuhan, China). After being washed with PBST, the membrane was incubated with a horseradish peroxidase-coupled secondary antibody (SA00001-2, 1:10,000, Proteintech, Wuhan, China). Lastly, enhanced chemiluminescence (ECL, Vazyme, Nanjing, China) was used to detect the bands. The intensity of each band was quantified with ImageJ software version 2.0.0 for analysis.

### 2.9 Metabonomic analysis

#### 2.9.1 Collection of serum samples

Serum samples from 50 healthy subjects (Control), 50 patients with early diabetic nephropathy (DN-E) and 50 patients with advanced diabetic nephropathy (DN-A) were collected from Jiangsu Provincial Hospital of Traditional Chinese Medicine. The samples were stored at −80°C for subsequent experiments.

#### 2.9.2 Preparation of serum samples

After the addition of 200 μL of extraction solution (methanol: acetonitrile = 1:1, precooled at −40°C, containing 0.1% formic acid and an isotopically labeled internal standard mixture), the samples were vortexed for 30 s and sonicated for 10 min in an ice-water bath, followed by subsiding at −40°C for 1 h. After centrifugation (15 min, 12,000 rpm, and 4°C), a 200 μL aliquot of the supernatant was transferred to an Eppendorf tube. Then, the supernatant was evaporated to dryness under a gentle stream of nitrogen and reconstituted in 50 μL of water containing 0.1% formic acid. After centrifugation (15 min, 12,000 rpm, and 4°C), the clear supernatant was subjected to UHPLC-MS/MS analysis.

#### 2.9.3 UHPLC-Q-TOF MS analysis

Non-targeted metabonomics was performed using SCIEX Exion LC Ultra Performance Liquid Chromatography (UHPLC) and a mixed quadrupole time-of-flight mass spectrometer (Triple TOF 5600^+^; AB Sciex, Boston, United States) to determine metabolic characteristics. An Atlantis TM premier BEH C18 ax (2.1 × 100 mm, 1.7 μM) with a VanGuardTM FIT was used to operate at 40°C. The mobile phase system consisted of 0.1% formic acid water (v/v, solvent A) and acetonitrile (solvent B), the flow rate was set at 0.4 mL/min, and the injection volume was 5 μL.

MS analysis was performed in ESI-positive (+)/negative (−) ion mode. The ESI source conditions were as follows: nitrogen was used as the atomizer and auxiliary gas, and the atomizing gas pressure was 1 (GS1) 55 psi; the auxiliary gas pressure was 2 (GS2) 55 psi; the curtain gas pressure was 35 psi; the ion source temperature was 550°C; and the spraying voltage was 5500 V (+)/−4500 V (−).

In the TOF MS-IDA-MS/MS acquisition, the spectral mass scanning range of TOF MS was 50∼1,000 m/z, and the accumulation time was set to 0.10 s/spectrum. The scanning mass range of the product ions was 40∼1,000 m/z, and the accumulation time was set to 0.05 s/spectrum. The parameters of IDA mode were set as follows: the de-clustering voltage was 80 V (+)/−80 V (−), the collision energy was 35 15 V (+)/−35 15 V (−), the ionic strength was not less than 100 cps, the isotopes within 4 Da were excluded, the ionic tolerance was 50 mDa, and 10 cycles of monitoring were performed at each cycle. mDa, and 10 candidate ions were monitored per cycle.

#### 2.9.4 UHPLC-MS/MS targeted amino acid metabolomics

UHPLC separation was performed using an Agilent 1,290 Infinity II series UHPLC System (Agilent Technologies, California, USA) equipped with a Waters ACQUITY UPLC BEH amide column (100 × 2.1 mm, 1.7 μm). Mobile phase A was 1% formic acid in water, and mobile phase B was 1% formic acid in acetonitrile. The column temperature was set at 35°C. The autosampler temperature was set at 4°C, and the injection volume was 1 μL.

An Agilent 6460 triple quadrupole mass spectrometer (Agilent Technologies, California, USA) equipped with an AJS electrospray ionization (AJS-ESI) interface was used for assay development. The typical ion source parameters were as follows: capillary voltage = +4000/−3500 V, nozzle voltage = +500/−500 V, gas (N2) temperature = 300°C, gas (N2) flow = 5 L/min, sheath gas (N2) temperature = 250°C, and sheath gas flow = 11 L/min, nebulizer = 45 psi.

#### 2.9.5 UHPLC-MS/MS targeted tryptophan metabolomics

UHPLC separation was performed using an ACQUITY Premier instrument (Waters, Milford, USA) equipped with a Waters ACQUITY UPLC HSS T3 column (100 × 2.1 mm, 1.8 μm, Waters). Mobile phase A was 0.1% formic acid in water, and mobile phase B was 0.1% formic acid in acetonitrile. The column temperature was set at 40°C. The autosampler temperature was set at 10°C, and the injection volume was 5 μL.

A SCIEX Triple Quad™ 6500^+^ mass spectrometer (AB Sciex, Boston, USA) equipped with an IonDrive Turbo V electrospray ionization (ESI) interface was used for assay development. The typical ion source parameters were as follows: curtain gas = 40 psi, ion spray voltage = ±4500 V, temperature = 500°C, ion source gas 1 = 30 psi, and Ion Drive ion source gas 2 = 30 psi.

#### 2.9.6 Multivariate data analysis and biomarker identification

The raw data obtained from Analyst^®^ 1.7.1 software (SCIEX) were quantified using MultiQuantTM software. After the 80% rule was used to delete missing values, the other missing values were replaced with 1/5 of the minimum positive value of each variable. Then, the original data were preprocessed by median normalization, Pareto scaling and logarithmic transformation to obtain clean data, and single-factor and multifactor statistical analyses were subsequently performed. All the steps were completed at https://www.metaboanalyst.ca/ 5.0. The preprocessed data are imported into SIMCA software (version 14.1; Umetrics) and MetaboAnalyst 5.0 for PLS-DA analysis. According to the VIP, P value, FDR and FC values, the metabolites with VIP > 1 and P value < 0.05 were considered to be significantly different; FC > 1 indicated that the metabolites were upregulated, and FC < 1 indicated that the metabolites were downregulated, so the metabolites with the same trend were screened. An ROC curve was used to analyze and quantify the results of the regression analysis, the AUC value was calculated, and the diagnostic rate of metabolites for diseases was expressed as the AUC value. GraphPad Prism 8 (GraphPad software, La Jolla, California, USA) was used to process the ROC curves. MetaboAnalyst 5.0 was used for pathway enrichment analysis, and significantly different pathways were identified.

### 2.10 Metabonomic analysis

The data are expressed as the means ± standard error (SE). Two-tailed t tests were used to analyze the data from both groups. The differences between groups were compared by one-way ANOVA. P < 0.05 was considered statistically significant.

## 3 Results

**FIGURE 1:**
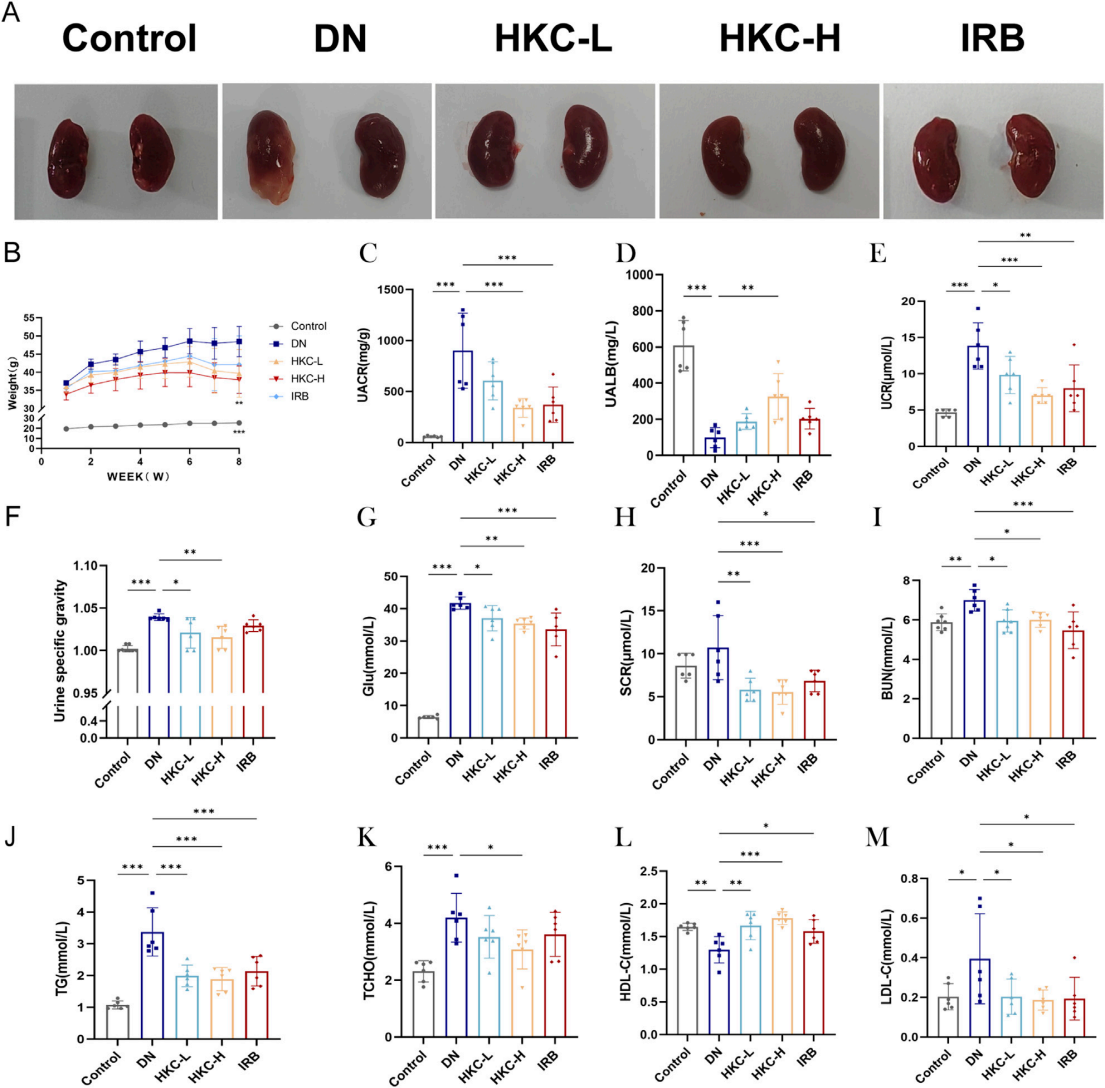
Effects of HKC on DN mice and analysis of blood and urine biochemical indexes. **(A)** Kidney morphology changes in mice; **(B)** Body weight changes in mice administered for 8 weeks; **(C)** Urinary albumin/creatinine ratio (ACR) level in urine; **(D)** Urinary albumin (UALB) level in urine; **(E)** Urinary creatinine (UCR) level in urine; **(F)** Specific gravity (SG) level in urine; **(G)** Blood glucose (Glu) level; **(H)** Serum creatinine (SCR) level; **(I)** Serum urea nitrogen (BUN) level; **(J)** Serum triglyceride (TG) level; **(K)** serum total cholesterol (TC) level; **(L)** serum high-density lipoprotein (HDL-C) level; **(M)** low-density lipoprotein (LDL-C) level. (^*^P < 0.05, ^**^P < 0.01, ^***^P < 0.001, n = 6).

To evaluate the therapeutic effect of HKC on the DN model induced in db/db mice, we collected urine, serum and kidneys from the mice. In the DN group, there were obvious pathological changes in the appearance and abscesses in the kidneys, and the administration of HKC effectively improved the pathological changes (Figure 1A). HKC treatment (1.50 g/kg/d) significantly reduced the indices of DN model mice, such as body weight, UACR level, urine specific gravity (SG), blood glucose (Glu), serum creatinine (SCR), urea nitrogen (BUN), total cholesterol (TC) and triglycerides (TG), increased the level of high-density lipoprotein cholesterol (HDL-C), decreased the level of low-density lipoprotein cholesterol (LDL-C), and improved renal injury by regulating blood glucose, renal function and blood lipid levels, indicating that HKC effectively improved the kidney damage in DN model mice, and reduced their blood glucose and hyperlipidemia levels (Figures 1B–M).

**FIGURE 2:**
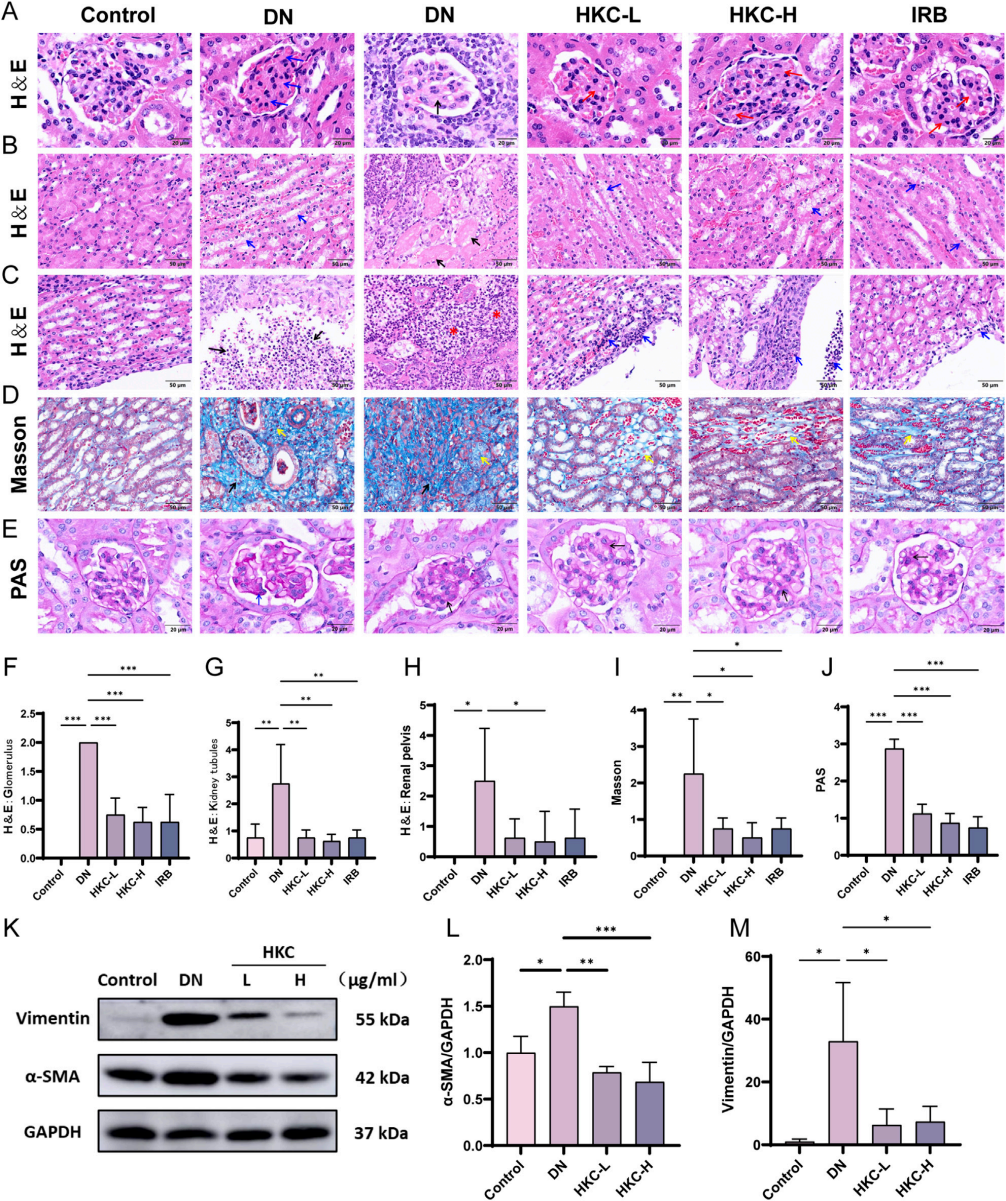
HKC ameliorates renal pathological injury in DN model mice. **(A)** HE staining to verify glomerular injury; **(B)** HE staining to verify renal tubular injury; **(C)** HE staining was used to verify the inflammatory injury in the renal pelvis; **(D)** Masson staining to verify glomerular injury; **(E)** PAS staining to verify glomerular basement membrane thickening; **(F)** Pathological score of glomerular injury (n = 4); **(G)** Pathological score of renal tubular injury (n = 4); **(H)** Pathological score of inflammatory injury in renal pelvis (n = 4); **(I)** Pathological score of glomerular injury (n = 4); **(J)** Pathological score of glomerular basement membrane thickening (n = 4); **(K)** Western blot analysis of α-SMA and Vimentin in mouse kidney; **(L)** Quantitative analysis of α-SMA (n = 3); **(M)** Quantitative analysis of Vimentin (n = 3). (^*^P < 0.05, ^**^P < 0.01, ^***^P < 0.001).

In the DN group, the red mesangial matrix was clearly increased in most glomeruli (blue arrow), In severe cases, mild glomerular fibrosis was found. (black arrow) (Figure 2A). Renal tubular epithelial cells were clearly edematous in the medulla (blue arrow), some severe renal tubular epithelial cells presented focal necrosis (black arrow) (Figure 2B). The mice presented obvious pyelonephritis, a large number of neutrophils infiltrated the renal pelvis (black arrow), and many neutrophils infiltrated the renal interstitium to form multiple renal abscess lesions (red asterisk). After treatment with HKC or IRB, the glomerular segmental mesangial matrix increased (red arrow) (Figure 2A). The number of edematous renal tubular epithelial cells decreased slightly (blue arrow) (Figure 2B). The cell infiltration of renal pelvis inflammation and renal interstitial inflammation was clearly weakened (blue arrow) (Figure 2C).

The Masson staining results revealed that the number of focal blue-stained collagen fibers in the DN group increased significantly (black arrow), and the number of interstitial local blue-stained collagen fibers increased in the medulla, suggesting that local fibrosis occurred in the low-dose Chinese medicine group, HKC treatment group and IRB group (yellow arrow) and that the range was significantly reduced (Figure 2D). PAS staining revealed that the matrix components in the DN group were increased (black arrow), the basement membrane was thickened (blue arrow), and the matrix was decreased after HKC treatment. These data revealed that HKC can effectively alleviate renal lesions, mesangial hyperplasia, renal tubular edema, fibrosis and pyelonephritis (Figures 2E–J). Subsequently, Western blotting was used to verify that the protein expression of α-SMA and vimentin in the kidney. In the DN group, the protein expression of these two proteins were significantly increased, while HKC treatment significantly reduced their expression, suggesting that HKC could effectively ameliorate renal fibrosis caused by DN (Figures 2K–M).

**FIGURE 3:**
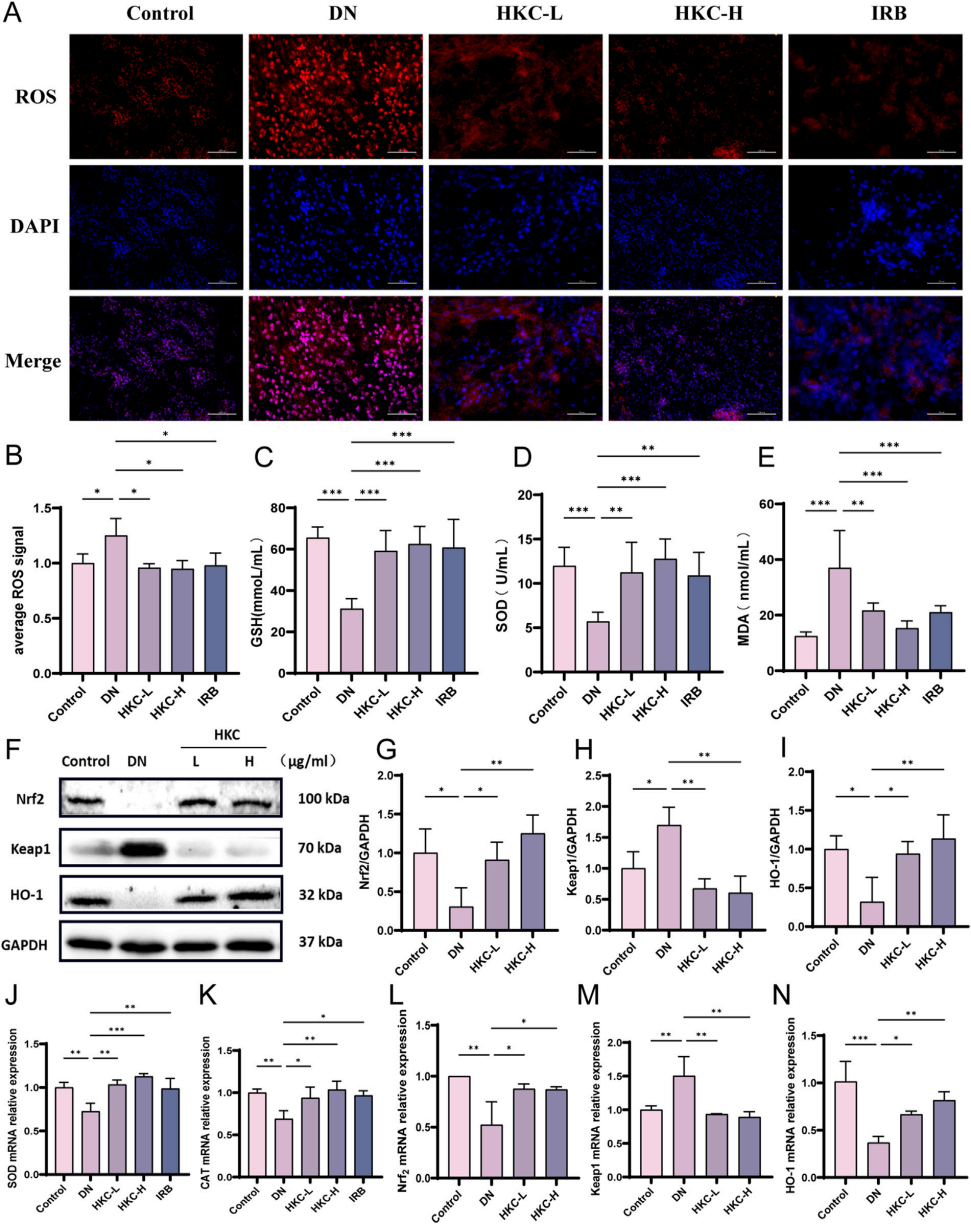
HKC ameliorates DN by regulating oxidative stress through Keap1/Nrf_2_/HO-1 pathway *in vivo*. **(A)** Fluorescence probe to detect the level of ROS in mouse kidney; **(B)** Quantitative analysis of ROS level in mouse kidney (n = 4); **(C)** GSH content in serum of mice (n = 4); **(D)** SOD content in serum of mice (n = 4); **(E)** MDA content in serum of mice (n = 4); **(F)** Western blot analysis of Nrf_2_, Keap1 and HO-1 in mouse kidney; **(G)** Quantitative analysis of Nrf_2_; **(H)** Quantitative analysis of Keap1; **(I)** Quantitative analysis of HO-1; **(J)** SOD mRNA expression in mouse kidney; **(K)** CAT mRNA expression in mouse kidney; **(L)** Nrf_2_ mRNA expression in mouse kidney; **(M)** Keap1 mRNA expression in mouse kidney; **(N)** HO-1 mRNA expression in mouse kidney. (^*^P < 0.05, ^**^P < 0.01, ^***^P < 0.001, n = 3).

We examined the fluorescence level of ROS in the kidneys of the mice by immunofluorescence, and found that the ROS in the DN group was significantly increased and there was obvious oxidative stress damage. Following treatment, the level of ROS was significantly reduced in the HKC-H group (Figures 3A,B). The GSH, SOD and MDA levels in the serum of the mice were detected by ELISA. The expression of GSH and SOD was upregulated, and MDA was downregulated after HKC treatment, which indicated that HKC treatment effectively improved the oxidative stress damage caused by DN (Figures 3C–E). Moreover, the Western blotting and mRNA expression results showed that HKC could upregulate the expression of Nrf_2_ and HO-1, downregulate the expression of Keap1. These results indicate that HKC regulate the Keap1/Nrf_2_/HO-1 pathway, enhance the antioxidant capacity and improve the kidney damage caused by DN (Figures 3F–N).

**FIGURE 4:**
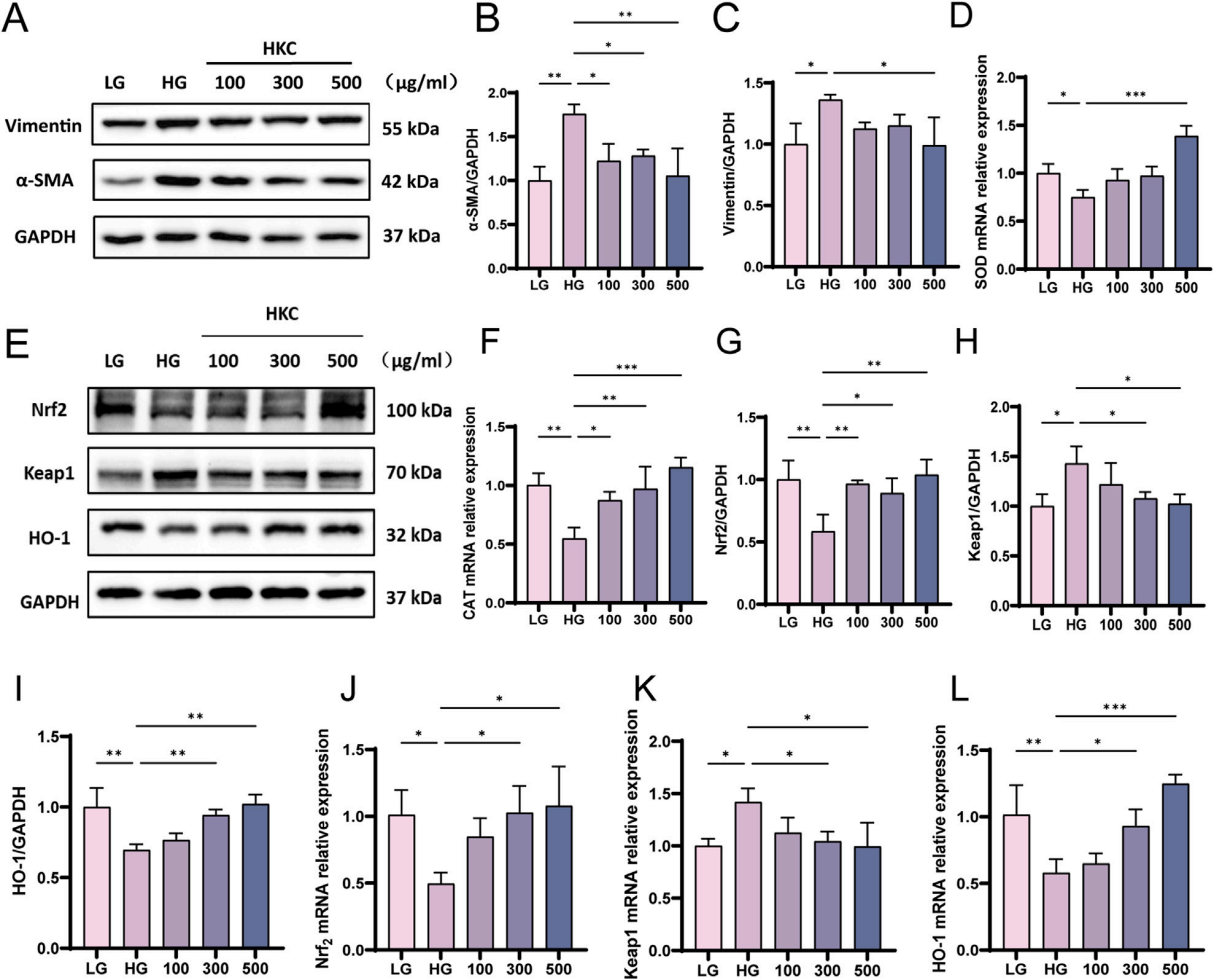
HKC ameliorates DN by regulating oxidative stress through Keap1/ Nrf2/HO-1 pathway *in vitro*. **(A)** Western blot analysis of α-SMA and Vimentin in HK-2 cells; **(B)** Quantitative analysis of α-SMA; **(C)** Quantitative analysis of Vimentin; **(D)** SOD mRNA expression in HK-2 cells. **(E)** Western blot analysis of Nrf2, Keap1 and HO-1 in HK-2 cells; **(F)** CAT mRNA expression in HK-2 cells. **(G)** Quantitative analysis of Nrf2 protein expression; **(H)** Quantitative analysis of Keap1 protein expression; **(I)** Quantitative analysis of HO-1 protein expression; **(J)** Nrf2 mRNA expression in HK-2 cells; **(K)** Keap1 mRNA expression in HK-2 cells; **(L)** HO-1 mRNA expression in HK-2 cells. (*P < 0.05, **P < 0.01, ***P < 0.001, n = 3).

Next, an injury model was established by mixing HK-2 cells with high concentrations of glucose. The Western blotting results verified the protein expression of α-SMA and vimentin, which proved that HKC could ameliorate renal fibrosis injury and renal tubular epithelial injury caused by HG in HK-2 cells *in vitro* (Figures 4A–C). The qPCR data verified that the HG caused oxidative stress injury, but after HKC treatment, the mRNA expression of SOD and CAT increased, and the level of oxidative stress improved (Figures 4D,F). Our data showed the protein levels of Nrf_2_ and HO-1 were increased, and the protein expression of Keap1 was decreased after HKC treatment, which were consistent with those *in vivo*. These results suggest that HKC can improve the renal injury and oxidative stress injury caused by high glucose through the Nrf_2_ pathway *in vitro* (Figures 4E,G–L).

Next, an non-targeted metabonomics analysis was employed to study the changes of serum metabolites in DN patients and healthy subjects (50 healthy subjects, 50 patients with early diabetic nephropathy and 50 patients with advanced diabetic nephropathy). It was found that the glomerular filtration rate (eGFR), BUN, SCR and UACR of DN-A patients were significantly higher than those of DN-E patients (Supplementary Table S2), indicating that the degree of renal injury in DN-A patients was significantly higher than that in DN-E patients. The levels of Glu and fasting C-peptide (FC-P) in DN-A patients were also significantly higher than those in DN-E, which also proved that diabetic nephropathy in DN-A patients was much more serious than that in DN-E patients (Supplementary Table S2).

**FIGURE 5:**
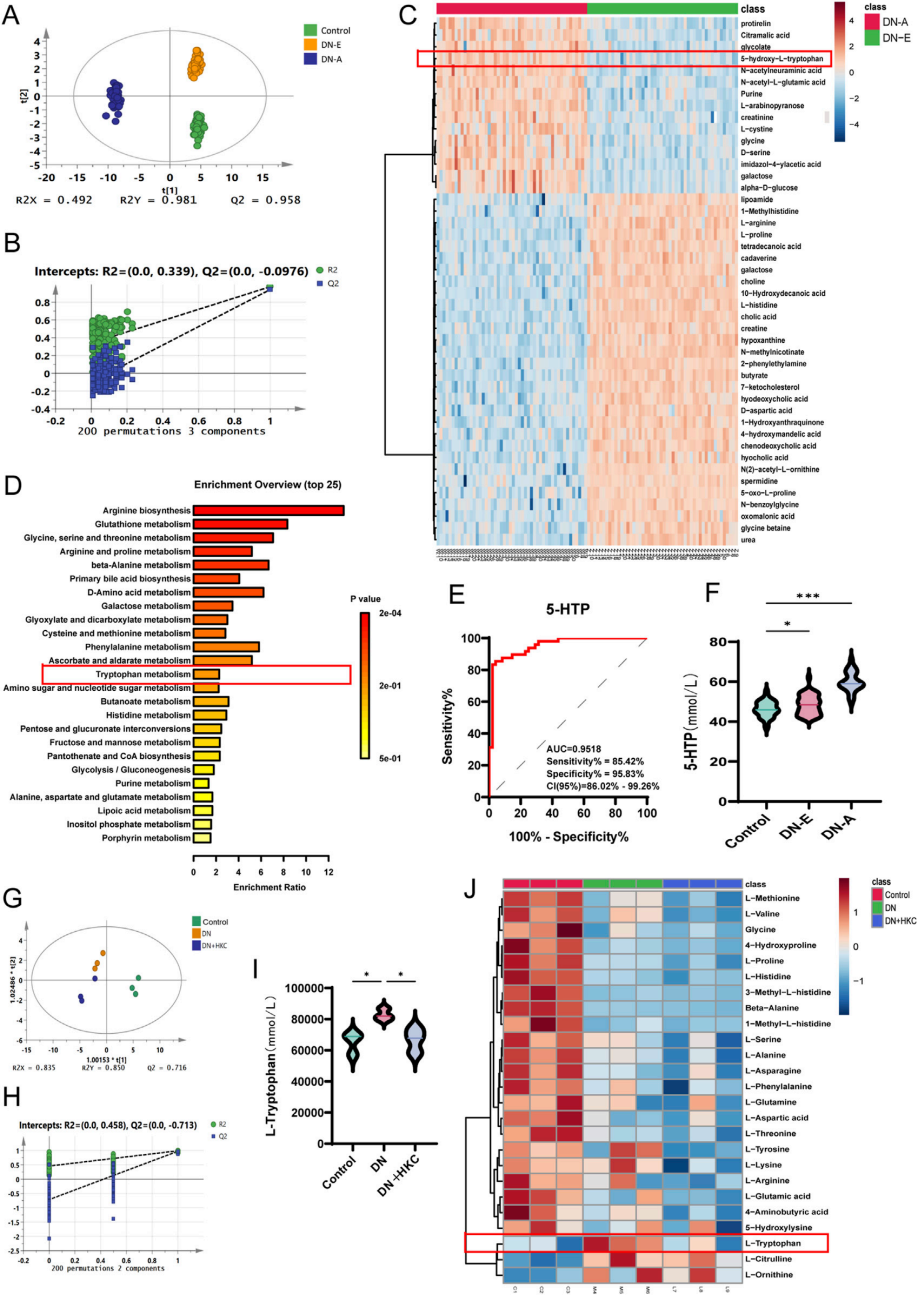
Targeted amino acid metabolomics analysis of clinical patient serum and non-targeted metabolomics analysis of mouse serum. **(A)** PLS-DA analysis of clinical non-targeted metabolomics (n = 50); **(B)** Permutation analysis of clinical non-targeted metabolomics; **(C)** heatmap analysis of clinical non-targeted metabolomics; **(D)** pathway enrichment analysis of clinical non-targeted metabolomics; **(E)** Receiver operating characteristic curve of 5-HTP in serum of clinical patients; **(F)** The level of 5-HTP in serum of clinical patients, **(G)** PLS-DA analysis of targeted metabolomics in mice (n = 3); **(H)** Permutation analysis of targeted metabolomics in mice; **(I)** L-tryptophan content in mouse serum; **(J)** Heatmap analysis of targeted metabolomics in mice.

The results of PLS-DA and permutation revealed that all the groups were well separated, with obvious differences between groups and good aggregation within groups. Compared with the Con group, the DN-E group and DN-A group had significantly different metabolites. Then, DN-E and DN-A were screened and analyzed, and 59 metabolites with significant changes were identified in the serum (Figures 5A,B). Heatmap analysis revealed significant changes in metabolites in the top 40 genes, revealing significant differences between DN-E and DN-A (Figure 5C), in which the 5-hydroxy-L-tryptophan (5-HTP) content gradually increased with the progression of the disease (Figure 5F). The area under the ROC curve (AUC) was 0.9518, and the specificity was 95.83%. The sensitivity was 85.42%, which shows that 5-HTP has good accuracy in the clinical diagnosis of diabetic nephropathy (Figure 5E). Pathway enrichment analysis revealed that the tryptophan metabolic pathway changed during the disease process (Figure 5D).

Late, the amino acid changes of different groups were analyzed by targeted metabonomics (n = 3). The PLS-DA and permutation results show the reliability of the three groups of data (Figures 5G,H). The heat map analysis revealed that among the targeted amino acids, tryptophan exhibited a significant metabolomic modulatory response to HKC intervention, and that the expression of tryptophan increased in the DN group but decreased after treatment with HKC (Figures 5I,J), indicating that tryptophan metabolism may be involved in the mechanism of HKC during the treatment of DN.

**FIGURE 6:**
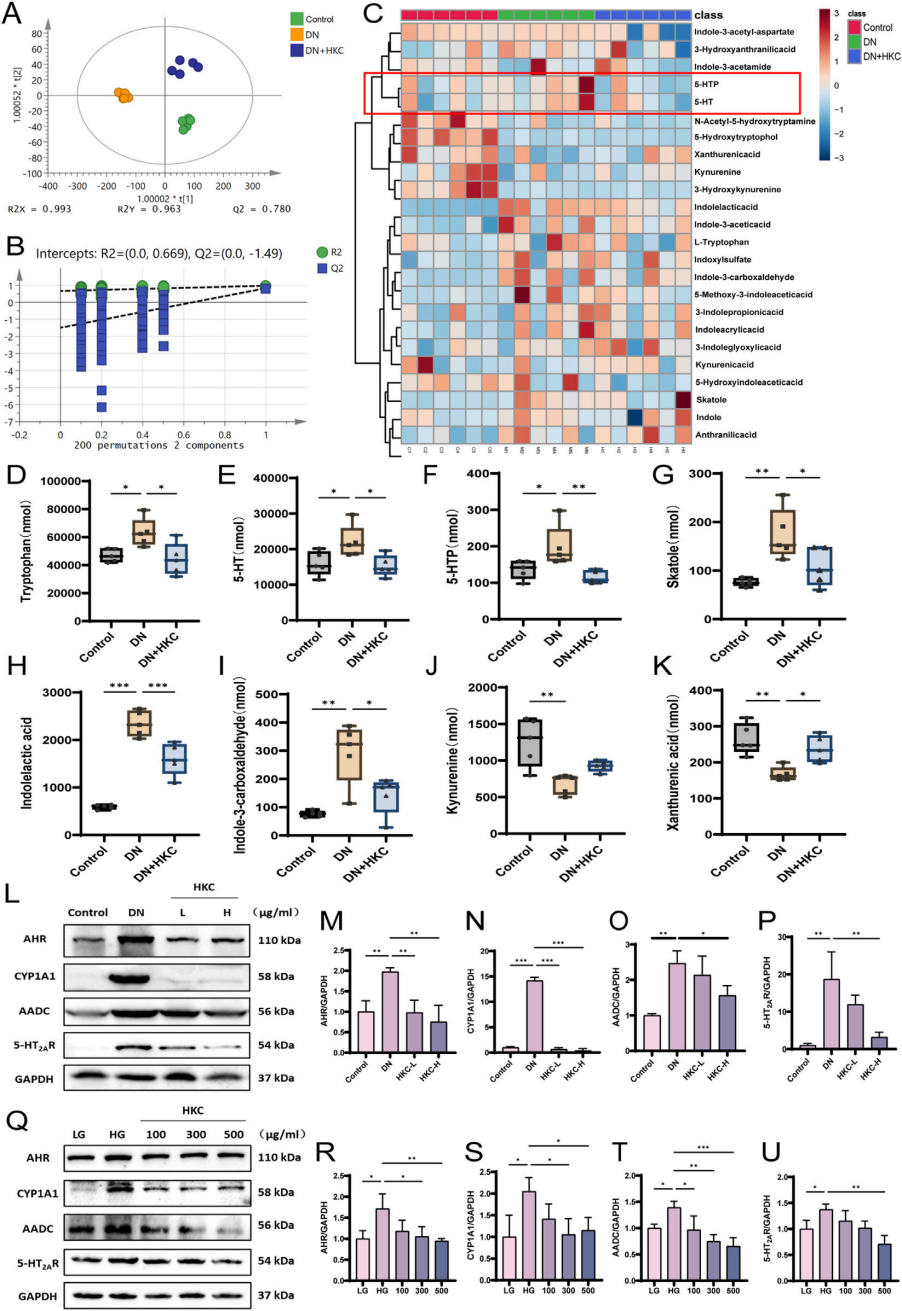
Targeted tryptophan metabolomics analysis of mouse serum and Western blotting verification of tryptophan metabolite-related protein expression *in vivo* and *in vitro*. **(A)** PLS-DA analysis of targeted tryptophan metabolomics in mice (n = 5); **(B)** Permutation analysis of targeted tryptophan metabolomics in mice; **(C)** Heatmap analysis of targeted metabolomics in mice; **(D)** Tryptophan content level; **(E)** 5-HT content level; **(F)** 5-HTP content level; **(G)** Skatole content level; **(H)** Indolelactic acid content level; **(I)** Indlole-3-carboxaldehyde content level; **(J)** Kynureninee content level; **(K)** Xanthurenic content level; **(L)** Western blot analysis of AHR, CYP1A1, AADC and 5-HT_2A_R in mouse kidney. **(M)** Quantitative analysis of AHR in mice; **(N)** Quantitative analysis of CYP1A1 in mice; **(O)** Quantitative analysis of AADC in mice; **(P)** Quantitative analysis of 5-HT_2A_R in mice; **(Q)** Western blot analysis of AHR, CYP1A1, AADC and 5-HT_2A_R protein in cells; **(R)** Quantitative analysis of AHR in cells; **(S)** Quantitative analysis of CYP1A1 in cells; **(T)** Quantitative analysis of AADC in cells; **(U)** Quantitative analysis of 5-HT_2A_R in cells. (^*^P < 0.05, ^**^P < 0.01, ^***^P < 0.001, n = 3).

The serum from the mice in the Control, DN and HKC-H groups was collected, and the changes in the expression of metabolites in the tryptophan metabolic pathway were analyzed by targeted tryptophan metabonomics (n = 5). The PLS-DA and permutation results show that the three groups of data have good intragroup aggregation and component separation (Figures 6A,B). Heatmap analysis revealed 24 metabolites, among which tryptophan, 5-HT, 5-HTP, skatole, indolelactic acid, and indole-3-carboxaldehyde increased in the model group but decreased after KHC treatment. (Figures 6C–K).

In addition, we verified the expression of tryptophan-related proteins and 5-HT pathway proteins *in vivo* and *in vitro*. The Western blotting results verified the protein expression of AHR, CYP1A1, AADC and 5-HT_2A_R. The expression of these proteins was significantly increased in DN mice and HK-2 cells in the HG model, and the expression of these proteins was significantly downregulated after HKC treatment. This effect may be significantly related to the metabolic expression of tryptophan in the model group, which stimulates the expression of AHR and its downstream CYP1A1 protein, and the 5-HT pathway is also activated by DN, which is inhibited after HKC administration, which were consistent with the above metabonomic results. Therefore, our data suggest that tryptophan metabolites may aggravate the renal lesions in DN and that HKC could improve DN by regulating tryptophan metabolism (Figures 6L–U).

**FIGURE 7:**
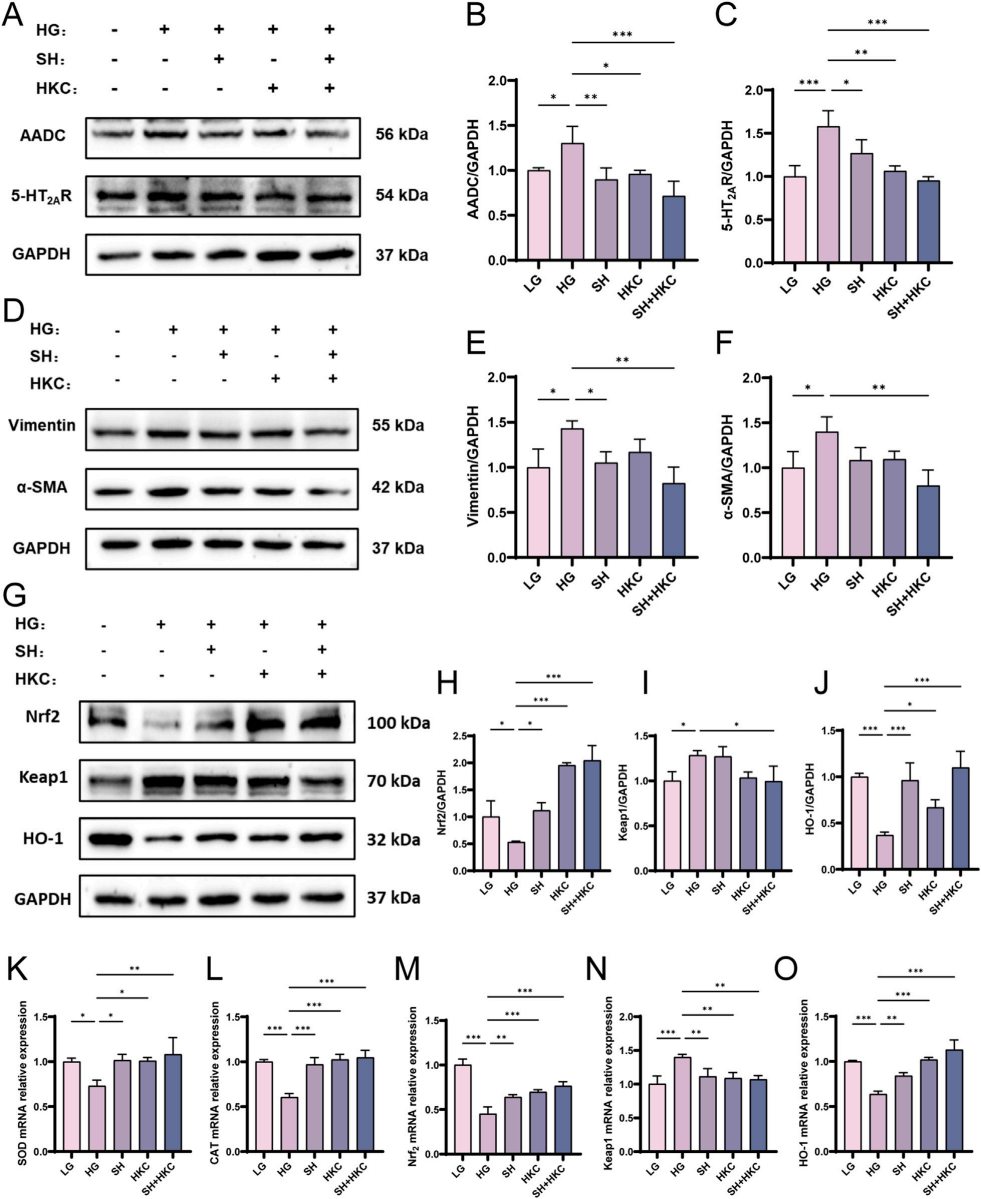
Sarpogrelate hydrochloride inhibits the pharmacodynamic effect of 5-HT in high glucose HK-2 Cells *in vitro*. **(A)** Western blot analysis of AADC and 5-HT_2A_R in HK-2 cells; **(B)** Quantitative analysis of AADC in cells; **(C)** Quantitative analysis of 5-HT_2A_R in cells; **(D)** Western blot analysis of α-SMA and Vimentin in HK-2 cells; **(E)** Quantitative analysis of α-SMA in cells; **(F)** Quantitative analysis of Vimentin in cells; **(G)** Western blot analysis of Nrf_2_, Keap1 and HO-1 in HK-2 cells; **(H)** Nrf_2_ quantitative analysis; **(I)** Quantitative analysis of Keap1 in cells; **(J)** HO-1 quantitative analysis; **(K)** SOD mRNA expression; **(L)** CAT mRNA expression; **(M)** Nrf_2_ mRNA expression; **(N)** Keap1 mRNA expression; **(O)** HO-1 mRNA expression. (^*^P < 0.05, ^**^P < 0.01, ^***^P < 0.001, n = 3).

The above results have revealed that HKC can improve DN by regulating the 5-HT pathway and the Keap1/Nrf_2_/HO-1 pathway. To verify the effect of 5-HT on diabetic nephropathy and the relationship between 5-HT and the Nrf_2_ pathway, we administered sarpogrelate hydrochloride to inhibit 5-HT in HK-2 cells, which were stimulated with HG combined with 500 μg/mL HKC for coadministration therapy. The results revealed that the expression of α-SMA and vimentin increased in response to HG and the expression of AADC and 5-HT_2A_R was significantly inhibited by SH (Figures 7A–F). SH could also effectively regulate the Keap1/Nrf_2_/HO-1 pathway, increase the expression of SOD and CAT, and this effect was greater when SH was used in combination with HKC (Figures 7G–O). These findings suggest that HKC can improve kidney injury induced by HG by influencing tryptophan metabolism and subsequently activating the Keap1/Nrf_2_/HO-1 pathway, increasing the expression of the antioxidant enzymes SOD and CAT.

## 4 Discussions

DN is considered a serious complication of diabetes and the main cause of end-stage renal disease, which has led to serious health problems and a considerable economic burden on human society worldwide. Patients with diabetic nephropathy usually display symptoms such as increased blood glucose and blood lipids, increased albuminuria levels and a decreased glomerular filtration rate (Jefferson et al., 2008), accompanied by various histopathological changes, such as thickening of the glomerular basement membrane, expansion of the extracellular matrix, podocyte injury and fibrosis. Pathological features such as vacuolar degeneration, loose arrangement and fibrosis can be observed in the tubulointerstitium (Jiang et al., 2019; Anders et al., 2018; Thomas et al., 2015). The commonly used drugs for DN in the clinic include renin-angiotensin system inhibitors (RASIs), such as angiotensin converting enzyme inhibitors (ACEIss) or angiotensin receptor blockers (ARBs), but their effects are not ideal for many clinical DN management practices (Doshi and Friedman, 2017; Tripathi and Yadav, 2013). Notably, traditional Chinese medicines have the characteristics of slow onset and multicomponent and multitarget synergy, so it may have more advantages in kidney protection. HKC have shown good effects in the prevention and treatment of DN, but their mechanism has not been thoroughly studied.

Oxidative stress (OS) plays a key role in DN. Hyperglycemia produces excessive ROS, which affects the main antioxidant protection mechanism (Behl et al., 2021). The antioxidation system is very important for maintaining redox homeostasis, which is primarily performed by enzymes such as thioredoxin (Trx), CAT, cytochrome c oxidase, SOD and glutathione peroxidase (GPx) (Hong and Park, 2021). In addition, nonenzymatic antioxidants such as GSH, vitamin C, vitamin E and carotenoids are also involved in antioxidant defense (Birben et al., 2012). These components antagonize ROS through various biochemical reactions. targeting different oxidant/antioxidant ratios, such as the Keap1/Nrf2/ARE ratio, is effective in preventing and controlling DN (Cheng et al., 2020). When an increase in the ROS level triggers the Keap1/Nrf_2_/ARE pathway, it will activate many genes and increase the production of many enzymes, including CAT, NAD(P)H: quinone oxidoreductase 1 (NQO1), SOD, HO-1 and GPx. These enzymes are effective in scavenging ROS and damaged cellular components from cells, and reducing inflammation and oxidative stress (Arellano-Buendía et al., 2016; Cheng et al., 2019; Tanase et al., 2022). In our study, we found higher levels of ROS, decreased expression of antioxidant enzymes such as SOD, CAT and GSH, and increased expression of MDA in the kidneys of db/db mice, suggesting the presence of oxidative stress in DN. By analyzing the mRNA and protein expression levels of Nrf_2_, Keap1 and HO-1, we demonstrated that HKC effectively improved the oxidative stress level in DN by regulating the Keap1/Nrf_2_/HO-1 pathway, which improved renal function in DN mice.

In addition, it has been found that the activity of the Nrf_2_ pathway is regulated by PTEN, and PTEN deficiency exacerbates renal inflammation and fibrosis in angiotensin II-induced hypertensive nephropathy, suggesting that PTEN affects renal pathological changes by regulating the infiltration and activation of medullary cells (Rojo et al., 2014; An et al., 2022). Nrf_2_ is also able to modulate the mRNA stability of STING to regulate STING, Nrf_2_ also regulates STING expression by modulating STING mRNA stability (Olagnier et al., 2018), and the cGAS-STING signaling pathway plays a key role in renal inflammation and fibrosis, and inhibition of cGAS-STING signaling attenuates renal inflammation and fibrosis in a model of unilateral ureteral obstruction (Jiao et al., 2025). Moreover, Keap1/Nrf_2_/HO-1 pathway has an important regulatory role in DN, so it can be hypothesized that PTEN and cGAS-STING signaling pathway may also have an important effect in DN, and together they can play a role in combating oxidative stress and inflammatory response to improve renal injury.

Metabonomics is an effective tool for studying the overall changes in metabolites in the body. Serum metabolomics can provide systemic metabolic information. However, there are differences in amino acid changes in the serum, urine and kidneys. For example, after kidney injury, renal tubular reabsorption decreases, glomerular permeability increases, and the amino acid content in the kidney decreases sharply, whereas the amino acid content in the urine and serum increases (Liao et al., 2022; Zhang et al., 2017). We first used non-targeted metabolomics to conduct an extensive screening of differential metabolites in normal subjects and DN patients, and firstly, the biochemical index information of the samples proved that compared to the Control group, the DN-A group did show significant elevated blood glucose and lipid levels and impaired renal function, which proved the reliability of the samples and could be relied upon as the accuracy of the results of non-targeted discovery metabolomics, and we obtained a large number of screening results for effective differential metabolites. The metabolites screened by serum metabolomics results in mice were also compared one by one, yielding differential metabolites with significant and identical changes in expression. It was further demonstrated that elevated tryptophan expression aggravated renal injury in DN patients and DN model mice. In addition, mouse-targeted metabolomics has deepened the regulation of tryptophan metabolism by HKC and explored the effects on the changes of tryptophan metabolites.

Tryptophan is an important aromatic amino acid. Key enzymes and metabolites in the tryptophan metabolic pathway have important roles in pancreatic islet function, insulin resistance, intestinal barrier, and angiogenesis, and dysregulation of tryptophan metabolism is closely related to the development of many complications, including DN (Gao et al., 2023). Tryptophan metabolism was highly expressed in the kidney, blood and urine metabolisms of mice with chronic nephropathy induced by cisplatin, and the tryptophan-related metabolite 5-hydroxy-indoleacetic acid was increased in the kidney, whereas tryptophan was decreased. The level of 5-hydroxy-indoleacetic acid (5-methoxy-indoleacetic acid) in the urine increased after cisplatin administration. These changes were reversed by HKC, indicating that tryptophan metabolism plays a key role in the process by which HKC reduces CKD (Liao et al., 2022). Recent studies have shown that tryptophan and its metabolites are important biomarkers of chronic and acute nephropathy. For example, the accumulation of indole sulfate, a tryptophan metabolite, is usually a sign of renal insufficiency. When the renal function is damaged, it accumulates, accelerates damage to renal tubular cells, and induces interstitial fibrosis and glomerulosclerosis (Tan et al., 2021; Devlin et al., 2016). The changes in tryptophan and its metabolites may indicate that renal injury is related to the regulation of oxidative stress, inflammation and the immune response (Song et al., 2021).

In the renal vascular system, 5-HT plays a direct and indirect role by regulating sympathetic renal innervation in normal blood glucose and diabetic animals (Morán et al., 2008). 5-HT_2_ receptors, which are widely expressed in renal tissue, mediate harmful effects, such as vasoconstriction, inflammation and fibrosis, which are the key processes involved in the development and progression of diabetic nephropathy (Yang et al., 2017b). Studies have shown that the serum concentration of 5-HT in diabetic patients is increased, and the pathogenesis of diabetic nephropathy involves 5-HT_2A_ receptor (5-HT_2A_) on renal mesangial cells (Kobayashi et al., 2008), which may be related to the production enhancement of type IV collagen by human mesangial cells induced by 5-HT in the kidney (Kasho et al., 1998). More importantly, SH can prevent diabetic nephropathy caused by diabetes (Doggrell, 2003b), improve renal function, inhibit renal vascular contraction induced by sympathetic nerves, reduce the increase in renal injury markers, improve renal hypertrophy (Fernández-González et al., 2024), inhibit the secretion of type IV collagen by human mesangial cells induced by 5-HT (Kasho et al., 1998), and improve oxidative stress induced by HG (Sun et al., 2011). The effect of 5-HT on the 5-HT_2A_R increases the synthesis of interleukin-6 in vascular smooth muscle, which may contribute to the activation of inflammation, and SH may also play an anti-inflammatory role (Ito et al., 2000). This study verified that the inhibition of 5-HT synthesis by SH *in vitro* can alleviate renal fibrosis injury and ameliorate oxidative stress injury.

Changes in tryptophan levels may reflect the deterioration of renal function and have the potential to serve as an alternative prognostic marker for DN (Chou et al., 2018). And it has been shown that tryptophan metabolites play a key role in chronic kidney disease by regulating the AHR signaling pathway. Miao et al. found that indole-3-aldehyde (IAld) significantly ameliorated renal fibrosis in CKD by inhibiting the AHR signaling pathway, as evidenced by the fact that IAld downregulates the expression of its downstream target genes (e.g., CYP1A1, CYP1B1) by reducing the nuclear translocation of the AHR, and thus inhibiting the production of pro-fibrotic proteins (e.g., α-SMA, collagen I) (Miao et al., 2024a). Similarly, indole metabolites produced by tryptophan metabolism were found to ameliorate renal injury by antagonizing AHR activity as well in a model of membranous nephropathy (MN) (Miao et al., 2024b). Moreover, a significant correlation has been found between dysbiosis of the gut microbiota and renal pathological injury (Shi et al., 2023), and a significant increase in urotoxins of intestinal origin (e.g., tryptophan metabolites such as indole sulfates) also leads to dysbiosis of the gut microbiota, which in turn exacerbates renal oxidative stress and fibrosis (Huang et al., 2024; Li et al., 2024; Van Mulders et al., 2025). These studies suggest that despite the varied etiology of different nephropathy types, over-activation of the action of tryptophan metabolites may be a common pathologic mechanism that is universal.

However, there are still some shortcomings in the article, such as the lack of *in vivo* studies of 5-HT_2A_ receptor inhibitors in DN mice in the experimental design, and in the subsequent verification of the mechanism is still not deep enough, and should be followed up by more experimental methods in the verification of the target through different directions.

## 5 Conclusion

**FIGURE 8:**
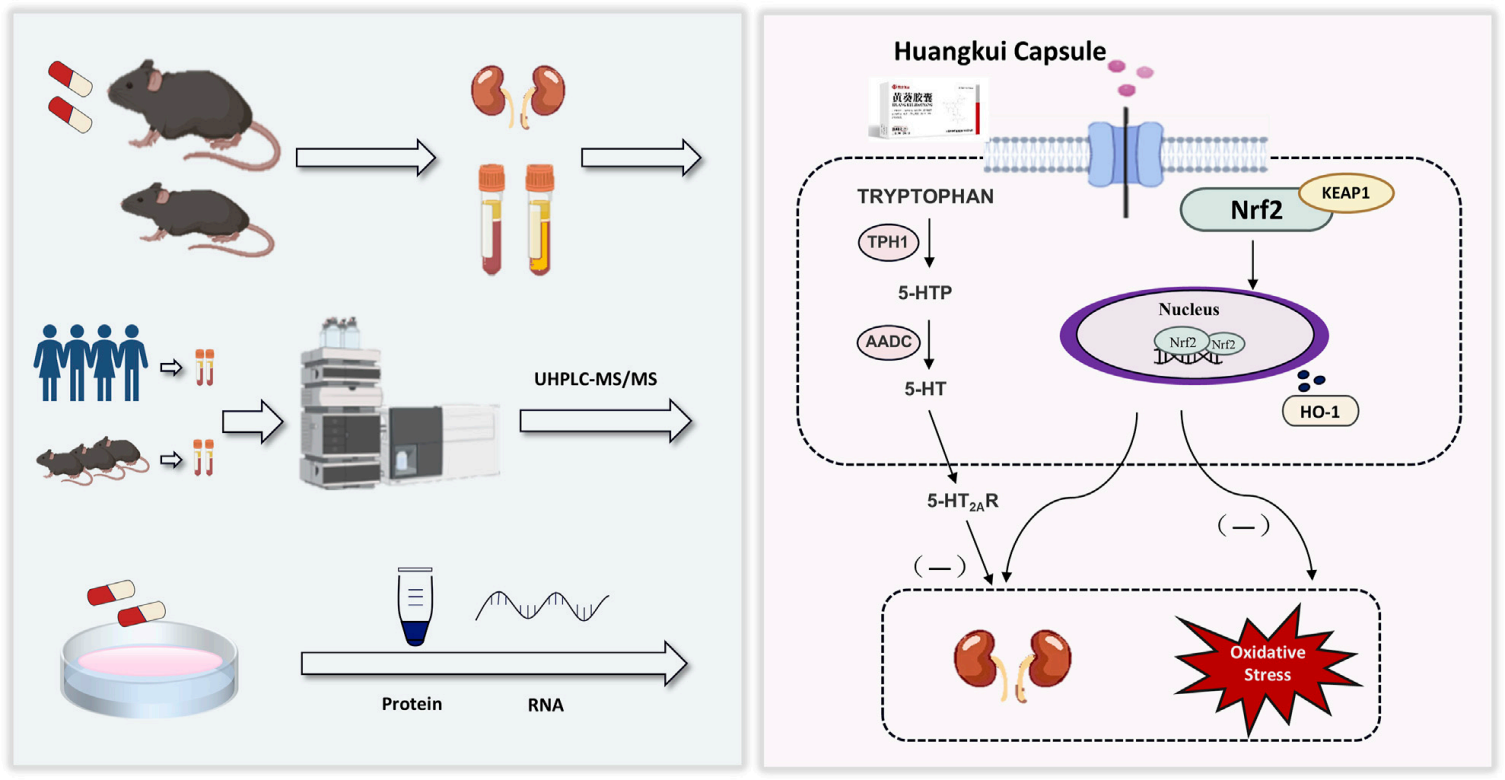
Workflow of the present study. We validated the effect of HKC on DN in animal and cellular models, and in combination with metabolomics found that HKC could regulate tryptophan metabolism and Keap1/Nrf_2_/HO-1 pathway to ameliorate renal fibrosis injury.

This study reveals the critical role of HKC in regulating tryptophan metabolism, particularly the 5-hydroxytryptamine (5-HT) pathway, through *in vivo* and *in vitro* experiments, supplemented by clinical metabolomic validation. HKC can effectively reduce the effects of 5-HT and ROS levels, and decrease the level of oxidative stress in DN, through the activation of the Keap1/Nrf_2_/HO-1 antioxidant pathway. Specifically, this effect contributed to the improvement of renal function in DN patients, including attenuation of renal fibrosis and tubular injury. In the future, it will be necessary to explore the specific role of 5-HT inhibitors in mouse models of DN and to identify specific targets for HKC in ameliorating diabetic nephropathy. The mechanism of HKC in the treatment of diabetic nephropathy is schematically illustrated in Figure 8.

## Data Availability

The datasets presented in this study can be found in online repositories. The names of the repository/repositories and accession number(s) can be found in the article/Supplementary Material.

## Abbreviations

DN, Diabetic nephropathy; HKC, Huangkui capsule; HE, Hematoxylin-eosin; PAS, Periodic acid-Schiff; ROS, Reactive oxygen species; HK-2, Human kidney-2; LG, Low-glucose; HG, High-glucose; 5-HT, 5-Hydroxy tryptamine/serotonin; 5-HTP, 5-hydroxy-L-tryptophan; Keap1, Kelch like ECH associated protein 1; Nrf2, Nuclear factor erythroid 2-related factor 2; HO-1, Heme oxygenase-1; UCAR, Urinary albumin/creatinine ratio; AADC, Aromatic amino acid decarboxylase; SOD, Superoxide dismutase; CAT, Catalase; MDA, Malondialdehyde; GSH, Glutathione; Glu, Blood glucose; TC, Total cholesterol; TG, Triglyceride; SCR, Serum creatinine; BUN, Blood urea nitrogen; HDL-C, High-density lipoprotein cholesterol; LDL-C, Low-density lipoprotein cholesterol; UALB, Urinary albumin; DN-E, Early diabetic nephropathy; DN-A, Advanced diabetic nephropathy; PCA, Principal components analysis; PLS-DA, Partial least squares Discriminant Analysis.
