# Supplementary material for: Huangkui capsules regulate tryptophan metabolism to improve diabetic nephropathy through the Keap1/Nrf2/HO-1 pathway
Source: Front Pharmacol. 2025 Apr 30;16:1535352. doi: 10.3389/fphar.2025.1535352 (PMC12075421; doi:10.3389/fphar.2025.1535352)
Supplement: Supplementary file 1 [file Table1.docx]

Supplementary Material

**Table S1**

Primer base sequence.

| **Species** | **Gene** | **Forward** | **Reverse** |
| --- | --- | --- | --- |
| Homo sapiens | SOD | GGTGGGCCAAAGGATGAAGAG | GGTGGGCCAAAGGATGAAGAG |
| Homo sapiens | CAT | TGGGATCTCGTTGGAAATAACAC | TCAGGACGTAGGCTCCAGAAG |
| Homo sapiens | Nrf2 | TTCCCGGTCACATCGAGAG | TCCTGTTGCATACCGTCTAAATC |
| Homo sapiens | Keap1 | CTGGAGGATCATACCAAGCAGG | GGATACCCTCAATGGACACCAC |
| Homo sapiens | HO-1 | AAGACTGCGTTCCTGCTCAAC | AAAGCCCTACAGCAACTGTCG |
| Homo sapiens | β-actin | GGCTGTATTCCCCTCCATCG | CCAGTTGGTAACAATGCCATGT |
| house mouse | SOD | CAGACCTGCCTTACGACTATGG | CTCGGTGGCGTTGAGATTGTT |
| house mouse | CAT | ATGACGCTGTGGCAGATTGTT | CCGCAAGGCGAGCATAGAT |
| house mouse | Nrf2 | CTGAACTCCTGGACGGGACTA | CGGTGGGTCTCCGTAAATGG |
| house mouse | Keap1 | CTGAACTCCTGGACGGGACTA | CGGTGGGTCTCCGTAAATGG |
| house mouse | HO-1 | AAGCCGAGAATGCTGAGTTCA | GCCGTGTAGATATGGTACAAGGA |
| house mouse | β-actin | GGCTGTATTCCCCTCCATCG | CCAGTTGGTAACAATGCCATGT |

**Table S2**

Characteristics of the study participants.

| **Characteristics** | **C, n=50** | **DKD-E, n=50** | **DKD-A, n=48** | **p value** |
| --- | --- | --- | --- | --- |
| **Sex (male/female)** | 34/16 | 33/17 | 36/14 | — |
| **BMI (kg/m2)** | 24.85±3.28 | 26.554.36 | 24.42±5.61 | — |
| **HbA1c (%)** | — | 8.93±2.21 | 7.92±2.04 | ns |
| **eGFR（mL/min/1.73m^2^）** | — | 83.10±23.16 | 40.47±27.59 | *** |
| **ALB (g/L)** | 46.37±2.18 | 42.34±4.23 | 31.15±4.49 | *** |
| **BUN (mg/dL)** | 4.85±0.97 | 6.81±2.32 | 13.8±6.24 | *** |
| **Scr (μmol/L)** | 74.41±12.9 | 84.02±30.48 | 223.4±168.95 | *** |
| **Glu (mmol/L)** | 5.08±0.44 | 8.51±4.01 | 6.59±2.78 | * |
| **UA（mg/dL）** | 332.55±83.43 | 376.39±99.22 | 430.42±100.1 | ** |
| **TC (mmol/L)** | 4.81±0.75 | 4.59±1.2 | 4.72±2.12 | ns |
| **TG (mmol/L)** | 1.39±0.72 | 2.18±1.29 | 2.14±1.89 | ns |
| **HDL-C (mmol/L)** | 1.48±0.26 | 1.21±0.21 | 1.19±0.28 | ns |
| **LDL-C (mmol/L)** | 2.66±0.55 | 2.54±0.79 | 3±1.72 | ns |
| **UACR（mg/g）** | — | 114.88±112.09 | 5793.6±2919.95 | *** |
| **FC-P（ng/ml）** | — | 2.11±1.65 | 2407.16±1920.93 | *** |

Values are expressed as mean standard ± deviation. ns P＞0.05, * P < 0.05, ** P < 0.01, *** P < 0.001. p values represent t Student’s t comparisons between DKD-E and DKD-A.

BMI=body mass index; HbA1c=Hemoglobin A1c; ALB=Albumin; BUN=Blood Urea Nitrogen; Scr=Serum Creatinine; Glu=Glucose; UA=Uric Acid; TC=Total Cholesterol; TG=Triglycerides; HDL-C =High-density Lipoprotein cholesterol; LDL-C = Low-density Lipoprotein cholesterol; UACR=urinary albumin to creatinine ratio; FC-P= Fasting plasma C-peptide.
